# Targeting of nucleo‑cytoplasmic transport factor exportin 1 in malignancy (Review)

**DOI:** 10.3892/mi.2021.27

**Published:** 2021-12-28

**Authors:** Sibel Özdaş, İpek Canatar

**Affiliations:** Department of Bioengineering, Faculty of Engineering Sciences, Adana Alparslan Türkeş Science and Technology University, Adana 01250, Turkey

**Keywords:** nuclear pore complex, exportin 1, nuclear export, exportin 1 inhibitor

## Abstract

Nuclear pore complexes (NPCs) regulate the entry and exit of molecules from the cell nucleus. Small molecules pass through NPCs by diffusion while large molecules enter and exit the nucleus by karyopherins, which serve as transport factors. Exportin-1 (XPO1) is a protein that is an important member of the karyopherin family and carries macromolecules from the nucleus to the cytoplasm. XPO1 is responsible for nuclear-cytoplasmic transport of protein, ribosomal RNA and certain required mRNAs for ribosomal biogenesis. Furthermore, XPO1-mediated nuclear export is associated with various types of disease, such as cancer, inflammation and viral infection. The key role of XPO1 in carcinogenesis and its potential as a therapeutic target has been demonstrated by previous studies. Clinical use of novel developed generation-specific XPO1 inhibitors and their combination with other agents to block XPO1-mediated nuclear export are a promising new treatment strategy. The aim of the present study was to explain the working mechanism of XPO1 and inhibitors that block XPO1-mediated nuclear export.

## 1. Nucleo-cytoplasmic transport

Nuclear pore complexes (NPCs) regulate the entry and exit of molecules from the cell nucleus. Small molecules (≤30 kDa) pass through NPCs by diffusion. However, larger molecules, such as RNA and proteins, enter and exit the cell nucleus via proteins called karyopherins, which are transport factors (1). Karyopherins (nuclear-cytoplasmic transport receptors family) denote a family of receptors associated with transport of molecules between the cytoplasm and nucleus of eukaryotic cells and comprise >19 members (including importins, exportins and transportins) (1,2). They are called karyopherins because they are found inside the nucleus in the karyoplasm (or nucleoplasm). Importins transport the cargo molecule from the cytoplasm to the nucleus, while exportins transport the cargo molecule from the nucleus to the cytosol. Moreover, transportins can transport molecules both from the nucleus to the cytoplasm and from the cytoplasm to the nucleus (3,4).

Karyopherins recognise and select the cargo molecule from its target sequences and transport it across the nuclear membrane (1,2). The nuclear target sequence is a short amino acid sequence, which enables the cargo molecule to be recognised by carrier karyopherins for entry and exit to the cell nucleus; it also determines the direction of transport (5). The nuclear target sequence recognised by importins when transporting the cargo molecule from the cytoplasm to the nucleus is referred to as the nuclear localization signal (NLS). The NLS sequence commonly comprises hydrophilic amino acids (especially lysine) (2,3). The target sequence that is recognized by the exportins when transporting the cargo molecule from the nucleus to the cytoplasm is called the nuclear export signal (NES) (2-4,6). The NES sequence is a short peptide of 10-15 amino acids with 3-4 regular leucine-rich hydrophobic amino acid repeats (generally conserved sequence Φ1-X_2,3_-Φ2-X_2,3_-Φ3-X-Φ4, where Φ is leucine, valine, isoleucine, phenylalanine or methionine and X is any amino acid) (7). Various mechanisms, such as post-translational modification (phosphorylation, acetylation, sumoylation), mutation or protein interaction, lead to changes in the intracellular location of the cargo molecule via the creation of new NES/NLS sequences, thus concealing the target sequence or changing the affinity of karyopherins to the cargo molecule (8,9).

The transfer of molecules larger than 30 kDa from NPCs, which cannot pass through the NPC by diffusion, is an energy-dependent process, and the function of importins and exportins is mediated by Ras-related nuclear protein (Ran). To perform their function, importins bind to Ran/GDP, whereas exportins bind to Ran/GTP (10). Exportins creates a cargo-exportin-Ran/GTP ternary complex in the nucleus by facilitating binding between cargo molecules and RanGTP. As this complex passes from the NPC to the cytosol, Ran/GTP is hydrolysed, Ran/GDP is formed and exportin releases the cargo molecule in the cytoplasm and then returns to the nucleus (11,12). By contrast, importins bind to RanGTP, releasing the cargo protein (2).

## 2. Structure of XPO1

In the human genome, exportins are encoded by six genes, one of the primary nuclear exporters is the Exportin-1 (XPO1) gene, also known as CRM1 (Ensembl no. ENSG000000828987). The XPO1 protein functions as a shuttle as a nuclear transport receptor between the cell nucleus and the cytoplasm (13,14). The XPO1 gene was discovered by Adachi and Yanagida in *Saccharomyces cerevisiae* and *Schizosaccharomyces pombe* cells in 1989. Mutations in the XPO1 gene first observed in mutant yeast strains deform the chromosome structure and the XPO1 gene is associated with preservation of chromosome structure. Therefore, the gene product has been named chromosome region maintenance 1 (CRM1) (15). The *S. pombe* XPO1 protein is homologous to the human protein originally named CC112. Given this homology, it became referred to as XPO1 instead of CC112(16). The XPO1 gene is localized in the 2p16 region of the second chromosome in humans (17). It consists of 60,778 bases organized into 25 exons and separated by 24 introns. This gene has 24 transcript variants. The XPO1 gene product is a 112 kDa protein. This mature polypeptide consists of 1,071 amino acids (13); it is a modular protein consisting of numerous functional domains and mediates the transport of ~220 proteins (18). Given that the N-terminal region of XPO1 protein (UniProtKB no. O149803) interacts with GTPase/Ran, it is believed that the affinity of its C-terminal region (707-1,034 residues) with cargo molecules is increased (19).

The C-terminal region (amino acids 707-1,027) of the CRM1 protein is composed of two U-shaped antiparallel α-helices and the HEAT repeat 15A-21A crystal structure was reported in 2004. In the Protein Data Bank, 26 data show the crystal structure of XPO1 alone or its interaction complexes with different proteins (20). Various methods, such as X-ray crystallography and electron microscopy, have revealed that XPO1 displays conformational flexibility as a transporter protein (21). They also shed light on the X-ray structure of the XPO1-RanGTP-Snurportin 1 and XPO1-RanGTP-RanBP1 (Ran binding protein 1) complexes. HEAT 9 interacts with the NES sequence of cargo proteins, serving as an allosteric inhibitor and controlling formation of the complex (22,23).

## 3. Physiological function of XPO1 in cells

**Figure 1:**
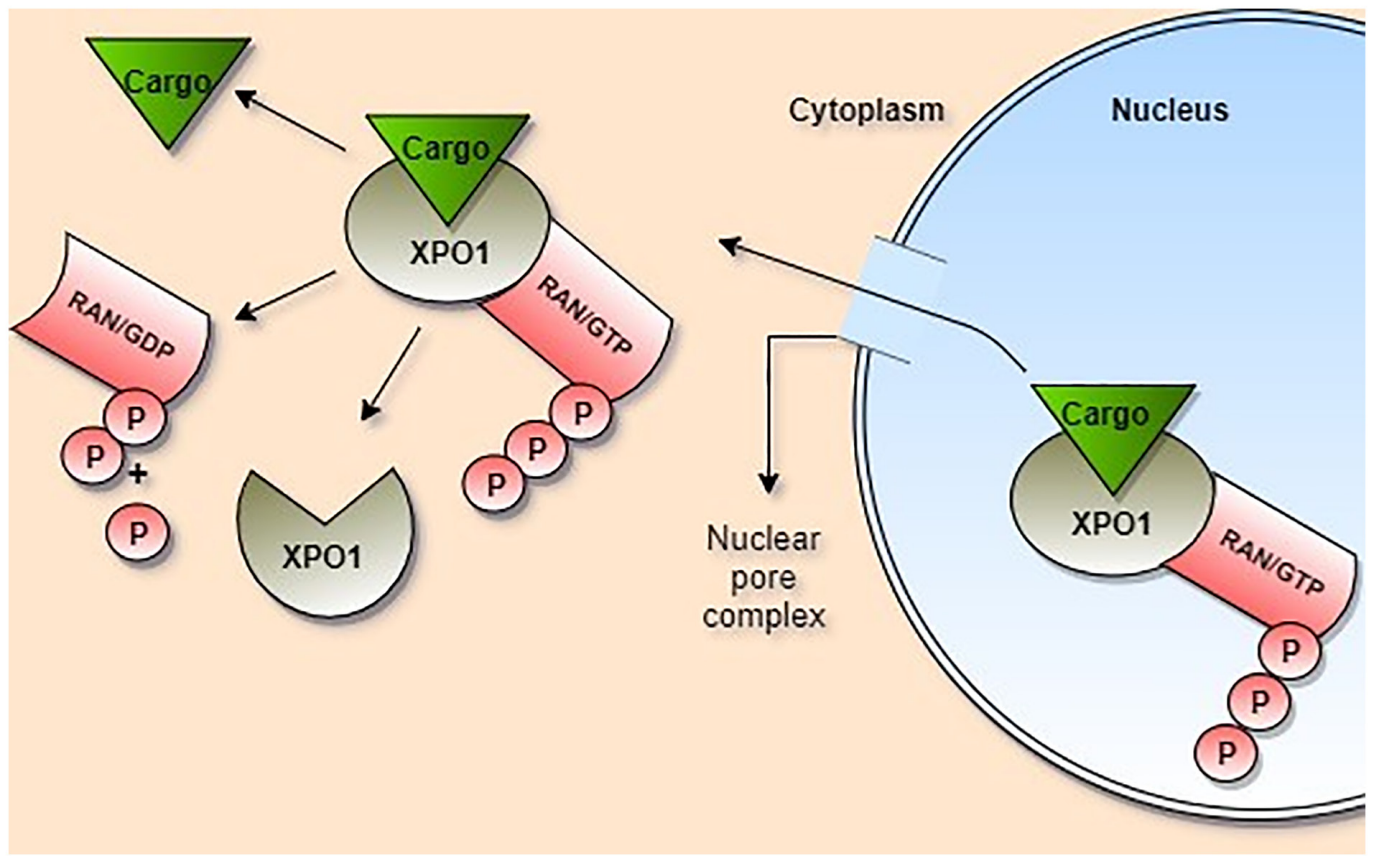
XPO1-mediated nuclear export. Ran/GTP and cargo bind to XPO1 and form a ternary complex. Afterwards, it passes through the nuclear pore complex. The affinity of XPO1 to the cargo molecule decreases because of hydrolysis of Ran/GTP to Ran/GDP in the cytoplasm. Subsequently, the cargo is released into the cytoplasm. XPO1, exportin 1; Ran, Ras-related nuclear protein; Phosphate, P.

XPO1 demonstrates weak binding with RanGTP in the nucleus and with the NES sequence of cargo molecules (24) However, when XPO1 simultaneously binds to RanGTP and to cargo molecules, its affinity for both increases by 500-1,000 times, and it passes from the NPC to the cytosol by forming a ternary complex (25). The hydrolysis of Ran/GTP to Ran/GDP in the cytoplasm decreases the affinity of XPO1 to cargo molecules by causing conformational changes in the protein structure, facilitating the release of cargo molecules (10-12) (Fig. 1). XPO1 and RanGDP pass through the NPC and return to the nucleus for a new transport cycle (2,25).

The human XPO1 gene is expressed in a cell cycle-dependent manner; mRNA transcription begins during the G_1_ phase of the cell cycle and increases during the G_2_/M phase (26). During interphase, the XPO1 protein is localized inside the nucleus near the nuclear membrane (26,27). High XPO1 expression levels have been observed in the brain, thymus, lung, spleen, liver, heart, pancreas, skeletal muscle, prostate, testis, placenta, ovary, small intestine, colon and peripheral blood leukocytes (14,19).

XPO1 mediates the transport of certain types of RNA, including viral/cellular mRNA, ribosomal RNA, transfer RNA and small nuclear RNAs (snRNAs), as well as various macromolecules, such as ribosomal subunit and NES-containing proteins rich in leucine, short peptide stretches containing hydrophobic residues, shuttle proteins, tumour suppressor proteins (TSPs), cellular or non-spliced or incomplete spliced RNAs of various viruses [such as human immunodeficiency virus (HIV)-1, human T-lymphotropic virus type-1 (HTLV-1) and influenza A] and HIV-1 Rev protein and HTLV-1 Rex protein that interact with Ran/GTP in the nucleus and cytosol (4,19,23,28-30).

## 4. XPO1 export of protein

In the proteomic analysis of yeast cells, 285 proteins regulated by XPO1 were detected, with ~45% of these containing known NES sequences (31). NES sequences for XPO1 comprise hydrophobic amino acids, including isoleucine, leucine, methionine, valine and phenylalanine (32). The NES sequences have a common conserved motif containing 10-15 amino acids with hydrophobic character [HX2-3HX2-3HXH, where H is a hydrophobic amino acid (such as isoleucine, leucine, methionine, valine and phenylalanine), X is any amino acid and the number is the potential number of repeats] (33). These hydrophobic amino acids form an α-helix or the entire loop structure, thus allowing XPO1 to attach to the hydrophobic pocket (25). However, NES sequences are yet to be sufficiently defined, as evidenced by the fact that <40% are known (7). The change in the cargo molecule's three-dimensional structure by mutation, phosphorylation and dephosphorylation results in the formation of a new NES to which XPO1 binds (8,34,35). It also causes NES sequences to be lost or masked or leads to the emergence of new sequences by additional modifications, including ubiquitination (33-35), acetylation (34), 10 sumoylation (9) or binding protein to specific cofactors such as RanGTP. Therefore, it alters the affinity of XPO1 to the cargo molecule (36,37).

Nuclear export of proteins, including STAT, NF-κB, nucleophosmin (NPM)1, Ras association domain family member SF2, Merlin, TSPs, such as APC, p53, p73, forkhead box O (FOXO), IKB, BCR-ABL, eukaryotic translation initiation factor 4E, BRCA1, viral proteins, regulatory/pro-inflammatory p21CIP, p27Kip1, retinoblastoma, anti-apoptotic proteins, such as NPM and AP-1 and oncogenic proteins, such as Cox-2, c-MYC, epidermal growth factor receptor and hypoxia inducible factor-1, is key for cell cycle and cell proliferation. In addition, their nuclear export is mediated by XPO1(38). Furthermore, XPO1 contributes to carcinogenesis by regulating the activity of TSPs and oncogenes. XPO1 controls multiple intracellular processes by regulating the localization of cyclin B, MPAK, MAPK-associated protein kinase 2, p21, p33, p27 and Survivin (39,40). Moreover, the overexpression of XPO1 leads to transport of Rev and U snRNA from the nucleus to the cytoplasm (13). It has been reported that the transcription factors nuclear transcription factor Y/CBP (CREB-binding protein), Sp1 and p53 are associated with the promoter of the XPO1 gene and serve an important role in the transformation of cancer cells by activating the promoter of XPO1(41). The XPO1 gene plays a role in the control of cell proliferation and affects the loss of control in cancer cell proliferation via various pathways. As a nuclear export factor, XPO1 regulates direct intracellular localization of cell cycle regulators, TSPs and pro-apoptotic proteins; therefore, the displacement of these proteins containing nuclear export sequences contributes to carcinogenesis and the development of drug-resistance mechanisms by regulating the activity of oncogenes.

## 5. Single nucleotide polymorphisms (SNPs) of XPO1

Genotypical variations in XPO1 may affect the function of the nuclear-cytoplasmic transport by altering expression levels of XPO1, resulting in emergence of certain disease phenotypes. The SNP c.1871A> G in XPO1 (pD624G) has been found to be associated with oesophageal squamous cell carcinoma and chronic lymphocytic leukaemia (42,43). Structural modelling study have shown that it is necessary to create a critical salt bridge with lysine at position 144 in the area where aspartic acid amino acid located at 624 of the XPO1 protein attaches to the target cargo molecule of XPO1. It has been reported that the conversion of the amino acid at the position 624 to glycine may cause the loss of this salt bridge and increase the nuclear-cytoplasmic transport efficiency by changing the affinity of XPO1 to the cargo molecule (42). SNP rs6735330 in XPO1 is associated with autism (44). In addition, SNP rs7600515 in XPO1 has been reported as a prognostic factor for Crohn's disease (45). SNP rs4430924 in XPO1 is also associated with the risk of developing hepatotoxicity of anti-tuberculosis drugs (46).

## 6. XPO1 in cancer

XPO1 is reported to be overexpressed in certain types of cancer (Table I).

In ovarian (47), cervical (48), glioma (49), osteosarcoma (50), pancreatic (51), oesophageal (52,53), lung (54), gastric (55), head and neck (56,57), renal cell carcinoma (58), hepatocellular carcinoma (59), acute lymphoid leukaemia (ALL) (60,61), chronic myeloid/lymphoid leukaemia (CML/CLL), multiple myeloma (MM) (62), mantle cell lymphoma (MCL) (63,64) plasma cell leukaemia (65), acute myeloid leukaemia (AML) (66) and breast cancer (67), an increase in the expression level of XPO1 was detected and it has been reported that this increase was associated with metastasis, increased tumour size, histological grade and decreased overall survival. The increased expression of XPO1 causes accumulation or mislocalisation of TSPs, cell cycle regulator and/or pro-apoptotic proteins in the cytoplasm with excessive nucleo-cytoplasmic transport, as well as deregulating ribosomal biogenesis, increasing carcinogenesis and development of resistance to chemotherapy (10). In addition, Crm1-dependent pathways serve a role in cancer pathogenesis since it is active in the control of mitosis and the dispersion of chromosomes and is important in maintaining and chromosome protected structure (68). One study reported that XPO1 overexpression in all solid tumour types except liver cancer and haematological malignancies (69). Higher XPO1 expression has been associated with worse patient prognosis in ovarian, pancreatic, oesophageal, thymic epithelial and breast tumour, as well as glioma. On the other hand, high XPO1 expression has been associated with a better prognosis in patients with osteosarcoma. Given XPO1 overexpression and its association with negative clinical outcome in various types of malignancy, it has become an attractive potential therapeutic target molecule in oncology (47,49-51,64).

## 7. Inhibitors of XPO1

It has been reported that suppressing XPO1-mediated nuclear export with specific agents or suppressing gene expression by XPO1-specific small interfering RNA activates apoptotic pathways and increases the sensitivity of tumour cells to chemotherapy drugs, such as doxorubicin, etoposide (70), cisplatin (71) and imatinib mesylate (72). Table II summarizes compounds that have been described as XPO1 inhibitors.

Numerous inhibitors of nuclear export derived from natural and synthetic sources have been identified (73). Leptomycin B (LMB), a natural compound, emerged as the first inhibitory molecule to block the function of the XPO1 protein (14,74). LMB is covalently bound at the specific cysteine (Cys528) residue located in the NES-binding cleft of the XPO1 protein; this inhibits binding of XPO1 to the target cargo molecule (74). LMB has been examined in multiple cancer cell lines and murine xenograft tumour models (75). Phase-I clinical LMB studies are continuing to investigate its use as an anti-cancer agent (70,75). However, LMB has not yet been used in clinical practice due to its low therapeutic index and high toxicity (76).

Other natural inhibitors of XPO1 include anguinomycins, isolated from *Streptomyces* species with selective cytotoxicity to transformed cells (77), and goniothalamin, obtained from *Goniothalamus macrophyllus.* Goniothalamin has been reported to induce G_2_/M phase cell cycle arrest and apoptosis in breast cancer cells (78,79). Moreover, 15d-PGJ2, a prostaglandin family member with both anti- and pro-inflammatory properties (80) and plumbagin, derived from *Plumbago zeylanica*, have been shown to have a suppressive role in XPO1 nuclear export function. In the presence of plumbagin, interactions between XPO1 and Foxo1, p21, p53 and p73 are disrupted (81). In particular, plumbagin serves an anti-tumour effect via suppression of nuclear export (82). Furthermore, piperlongumine, an alkaloid of the long pepper, induces nuclear retention of major TSPs including Foxo1, p53, p21 and IкB-α and blocks interactions between XPO1 and these proteins (83,84). The natural inhibitor ratjadon, isolated from *Sorangium cellulosum*, has anti-proliferative effects (85). In a previous study, ratjadon exerted anti-HIV activity (86). In one study, ratjadon was conjugated to small-molecule targeted ligands, which induced the inhibition of nuclear export in extracellular targeted cancer therapy. These conjugates retain their inhibitory activity by binding to XPO1(87).

These aforementioned natural inhibitors with molecular mechanisms similar to LMB bind covalently to Cys528 in the reactive XPO1 region. XPO1-mediated nuclear export is inhibited by such binding, resulting in nuclear accumulation of TSPs and growth regulatory proteins (GRPs) (88,89).

Contrary to the other natural XPO1 inhibitors, curcumin, a natural polyphenol product, also suppresses p53 nuclear export (90). Curcumin has various anti-inflammatory, antimicrobial, antioxidative and anti-cancer properties. Phase I/II studies of curcumin in multiple myeloma (MM) are ongoing (91).

Besides natural inhibitors, several synthetic compounds are also available to inhibit XPO1, including selective inhibitors of nuclear export (SINEs), KOS-2464, (R)-4'-methylklavuzon, CBS9106, Compound S109, PKF050-638 and Compound 1l. SINE compounds are produced based on an *in silico* molecular modelling strategy (82-95). SINEs are covalently bound to Cys528 residue of XPO1 and hinder XPO1 binding to target cargo molecules (92). In *in vivo* studies, oral use of SINEs was observed to cause mild gastrointestinal symptoms (93). The potential of SINEs to inhibit XPO1-mediated nuclear export has been demonstrated. Apoptosis of cancer cells and arrest of cells in the G_1_ phase of the cell cycle are induced by SINEs (62,93). The effectiveness of KPT-330 (selinexor), SINE compound, has been evaluated in clinical studies of solid tumours and haematological malignancy. The results of these clinical trials have shown it to be a promising therapeutic candidate (10,94). KPT-330 has been found to have positive effects in clinical studies of hematopoietic malignancy, such as MM, AML and non-Hodgkin lymphoma (NHL). KPT-330 has been subjected to phase I/II studies in patients with AML (92,95). Notable decreases in tumour size have been demonstrated in a preclinical animal study with KPT-330. It has been demonstrated that KPT-330 has high efficacy in combination with various standard therapies, including selinexor/doxorubicin and selinexor/dexamethasone combinations (96,97). Moreover, a decrease in XPO1 levels was observed in studies conducted with other SINEs, including KPT-185 and KPT-251(98). Thus, the nuclear localisation of tumour suppressors may be preserved. Therefore, preclinical studies of XPO1 inhibition using SINE compounds may lead to a novel treatment for various types of cancer, including breast cancer (18,97,99). In addition, activity of KPT-185 and KPT-276 was investigated in NHL using *in vitro* and *in vivo* study. A high level of anti-tumour activity was observed in mouse models in which KPT-276 was orally applied. Therefore, KPT-276 is a promising candidate for NHL treatment (100).

Another synthetic XPO1 inhibitor is (R)-4'-methylklavuzon, which can retain tumour suppressor proteins in the nucleus by inhibiting the XPO1 protein. (R)-4'-methylklavuzon has been shown to be a novel drug candidate for treating hepatocellular carcinoma (101). KOS-2464, another synthetic molecule, is the most effective LMB analogue and has been reported to induce apoptosis at low nanomolar concentrations. Low toxicity and high anti-tumour activity of KOS-2464 have been demonstrated in various cancer cell lines and xenograft mouse models (33,59).

CBS9106 binds to the XPO1 reactive site, causing degradation, and its anti-tumor activity has been demonstrated in *in vitro* in various cancer cell lines and *in vivo* in xenograft animal models (33). Compound S109 is a derivative of CBS9106 that causes cell cycle arrest of large TSPs mediated by XPO1. Its anti-tumour activity has been investigated in colorectal and kidney cancer cells and it has been proven to inhibit proliferation and induce cell cycle arrest in these cells (84,85,102). Furthermore, PKF050-638 is a XPO1-inhibitor used in HIV treatment to inhibit the nuclear export of HIV-1 Rev protein; however, its anti-cancer effect has not been investigated yet (33,103). PKF050-638 interacts with cysteine in the NES-binding groove and prevents binding of the NES, similar to the mechanism of LMB (73). (R)-6-[(2-isopropyl-5-methylphenoxy) methyl]-5,6-dihydro-2-Pyron (compound 11) is a colourless oily liquid. The cytotoxic effects of compound 1l in HGC27 and MGC803 gastric cancer cell lines have been investigated; compound 1l was reported to degrade XPO1, inducing apoptosis in both MGC803 and HGC27 cell lines, exhibiting strong cytotoxic and anti-tumour effects against these cells (104).

## 8. Conclusion and future directions

Studies have demonstrated the key role of XPO1 in carcinogenesis and its potential as a therapeutic target (10,69). It has thus become the focus of efforts to develop new tumour treatment strategies. The clinical use of novel specific XPO1 inhibitors and their combination with other agents is promising. Suppressing gene expression with specific inhibitors or interference techniques have identified the biological function and intracellular mechanisms of XPO1 in malignancy, as well as drug resistance. Targeting XPO1 offers advantages in treatment strategies by activating various apoptotic pathways to avoid the development of drug resistance.

## Figures and Tables

**Table I:** Cancer types associated with exportin 1.

Cell line	Study design	Cancer	(Refs.)
OVCAR-3, SKOV-3, CAOV-3, ES-2, A2780, Mdah2744, OAW42, EFO21, EFO27, FU-OV-1, PA-1	*In vitro*	Ovarian	(47)
HeLa (HPV18), SiHa (HPV16), CaSki (HPV16 and 18), ME180, MS751 (HPV18), C33A	*In vitro*	Cervical	(48)
U251, SHG-44 U118, U87	*In vitro* and *in vivo*	Glioma	(49)
U2OS, HOS, Saos2, MG63	*In vitro*	Osteosarcoma	(50)
MiaPaCa-2, HPAC, AsPC-1, PANC-1	*In vitro* and *in vivo*	Pancreatic	(51)
WHCO1, WHCO5, WHCO, ECA109, TE1, TE8, KYSE306	*In vitro* and *in vivo*	Esophageal	(52,53)
A549, H460	*In vitro*	Lung	(54)
NCI-N87, SNU-1, SNU-16	*In vitro* and *in vivo*	Gastric	(55)
UT-SCC-16A, UT-SCC-16B, UT-SCC-60A, UT-SCC-60B, UT-SCC-74, UT-SCC-74B,	*In vitro*	Head and neck	(56,57)
e ACHN, Caki-1, 786-O	*In vitro* and *in vivo*	Renal cell carcinoma	(58)
SK-HEP-1, Huh7, MHCC97H, SNU-182, SNU-387, HepG2	*In vitro* and *in vivo*	Hepatocellular carcinoma	(59)
Plat-E, 293, OCI-AML-3, MOLM-13, and MV4	*In vitro*	Acute myeloid/lymphoid leukemia (AML/ALL)	(60,61)
HS-5	*In vitro* and *in vivo*	Chronic myeloid/lymphoid leukemia	(62)
HS-5	*In vitro* and *in vivo*	Multiple myeloma	(63)
JVM-2, Maver-1, NCEB-1, Jeko-1, DBsp53, Granta519, JVM-13, Z-138, Rec-1	*In vitro* and *in vivo*	Mantle cell lymphoma	(64,65)
MM1S, MM1R, INA6, INA6GFP, ANBL6 KMS18, KMS20	*In vitro* and *in vivo*	Plasma cell leukemia	(66)
RPMI8226, MOLP8, 28BM, 12PE, XG1, U266			
K562	*In vitro*	Acute myeloid leukemia	(67)

**Table II:** Exportin 1 nuclear export inhibitors.

Inhibitor	Compound	(Refs.)
Leptomycin B	Antibiotic	(14,74-76)
Anguinomycins	Antibiotic	(77)
Goniothalamin	Natural	(78,79)
15d-PGJ2	Natural	(80)
Plumbagin	Natural	(81,82)
Piperlongumine	Natural	(83,84)
Ratjadon	Antibiotic	(85,86,87)
Curcumin	Natural	(90,91)
SINE	Synthetic	(92-100)
(R)-4'-methylklavuzon	Synthetic	(101)
KOS-2464	Synthetic	(33,59)
CBS9106	Synthetic	(33)
Compound S109	Synthetic	(84,85,102)
PKF050-638	Synthetic	(33,73,103)
Compound 1l	Synthetic	(104)

## Data Availability

Not applicable.

## Authors' information

Sibel Özdaş, ORCID no. 0000-0003-4610-2785; İpek Canatar, ORCID no. 0000-0001-9448-8112.
